# Use of T1‐weighted/T2‐weighted magnetic resonance ratio images to elucidate changes in the schizophrenic brain

**DOI:** 10.1002/brb3.399

**Published:** 2015-09-25

**Authors:** Jun Iwatani, Takuya Ishida, Tomohiro Donishi, Satoshi Ukai, Kazuhiro Shinosaki, Masaki Terada, Yoshiki Kaneoke

**Affiliations:** ^1^ Department of NeuropsychiatryGraduate School of Wakayama Medical University811‐1 KimiideraWakayama641‐8509Japan; ^2^ Department of System NeurophysiologyGraduate School of Wakayama Medical University811‐1 KimiideraWakayama641‐8509Japan; ^3^ Wakayama‐Minami Radiology Clinic870‐2 KimiideraWakayama641‐0012Japan

**Keywords:** magnetic resonance imaging, myelin, nucleus accumbens, schizophrenia, T1‐weighted/T2‐weighted ratio, ventral putamen

## Abstract

**Introduction:**

One leading hypothesis suggests that schizophrenia (SZ) is a neurodevelopmental disorder caused by genetic defects in association with environmental risk factors that affect synapse and myelin formation. Recent magnetic resonance imaging (MRI) studies of SZ brain showed both gray matter (GM) reduction and white matter (WM) fractional anisotropy reduction. In this study, we used T1‐weighted (T1w)/T2‐weighted (T2w) MRI ratio images, which increase myelin‐related signal contrast and reduce receiver‐coil bias.

**Methods:**

We measured T1w/T2w ratio image signal intensity in 29 patients with SZ and 33 healthy controls (HCs), and then compared them against bias‐corrected T1w images.

**Results:**

Mean T1w/T2w ratio signal intensity values across all SZ GM and WM voxels were significantly lower than those for the HC values (analysis of covariance with age, gender, handedness, and premorbid intelligence quotient as nuisance covariates). SZ mean WM T1w/T2w ratio values were related to Global Assessment of Functioning (GAF) scores and were inversely related to the positive psychotic symptoms of the Positive and Negative Syndrome Scale. Voxel‐based analysis revealed significantly lower T1w/T2w ratio image signal intensity values in the right ventral putamen in SZ GM. T1w image intensities did not differ between the SZ and HC groups.

**Conclusions:**

T1‐weighted/T2‐weighted ratio imaging increased the detectability of SZ pathological changes. Reduced SZ brain signal intensity is likely due to diminished myelin content; therefore, mapping myelin‐related SZ brain changes using T1w/T2w ratio images may be useful for studies of SZ brain abnormalities.

## Introduction

Schizophrenia (SZ) is now considered to be a disorder of both structural and functional disconnectivity (Friston 1998; Shenton et al. 2001; Davis et al. 2003; Meyer‐Lindenberg et al. 2005; Haroutunian et al. 2014). Postmortem and genetic studies suggest impairment of myelination in the SZ brain (Hakak et al. 2001; Flynn et al. 2003; Tkachev et al. 2003, 2007; Sugai et al. 2004; Uranova et al. 2004). In vivo neuroimaging studies, such as diffusion tensor imaging (DTI), revealed white matter (WM) microstructural alterations that partly reflect myelin abnormalities in SZ (Kubicki et al. 2002, 2007; Ellison‐Wright and Bullmore 2009; Kubota et al. 2013; Lee et al. 2013). Myelination plays an important role in spike synchrony by modulating the neural signal conduction time (Lang and Rosenbluth 2003); thus, myelin impairment could lead to abnormal neural synchrony in SZ (Uhlhaas and Singer 2010). Such neural synchrony dysfunction would cause cognitive dysfunction and various symptoms in SZ (Uhlhaas and Singer 2010; Whitford et al. 2012). Studies on SZ gray matter (GM) using voxel‐based morphometry analyses revealed reduced volume and cortical thickness in various regions, including the prefrontal and temporal cortices (Kuroki et al. 2006; Ellison‐Wright et al. 2008; Glahn et al. 2008; Goldman et al. 2009; Schultz et al. 2010; Kubota et al. 2011). Furthermore, abnormalities in both GM and WM may be interrelated (Douaud et al. 2007; Koch et al. 2013; Sasamoto et al. 2014), which might be induced by signaling abnormalities that are genetically associated (Bennett 2011; Roussos and Haroutunian 2014).

Taken together, myelin‐related WM alterations and interrelated GM abnormalities in SZ suggest that myelin‐related GM alterations could also play an important role in SZ neuropathological processes. However, to the best of our knowledge, no GM myelin abnormalities have been reported in the SZ brain. Thus far, postmortem GM studies of the SZ brain found: (1) elevated neural density in the dorsolateral prefrontal cortex (Selemon et al. 1998); (2) the loss of dendritic spines on cerebral neocortical pyramidal neurons (Garey et al. 1998); (3) reduced cortical neuropil in the frontal and temporal cortices (Selemon and Goldman‐Rakic 1999); and (4) increased microglial numerical density in frontal area 9 (Rajkowska et al. 1998; Selemon et al. 2003; Garey 2010). However, those postmortem studies were limited only to specific SZ‐related brain regions (e.g., prefrontal cortex and temporal cortex), and did not study whole‐brain GM pathological changes.

It was recently proposed that the ratio of T1‐weighted (T1w) and T2‐weighted (T2w) magnetic resonance image (MRI) signal intensity can increase the sensitivity to detect myelin‐related signal intensity changes (Glasser and Van Essen 2011). They also showed that the GM ratio image corresponded well to subcortical WM myelination. We applied this method to spinal cord MRI, and reported that the contrast between GM and WM increased up to twofold, and an age‐related signal intensity change (probably due to myelin reduction with age) could be detected (Teraguchi et al. 2013). Furthermore, (Grydeland et al. 2013) applied this method to healthy control (HC) subjects, and found that intracortical myelin increased up until the 30 sec, followed by relative stability for years before declining in the 50 sec, and that this was related to cognitive performance. Recently, several improved methods were presented that used the ratio of T1w and T2w MRI images (Ganzetti et al. 2014; Shafee et al. 2015). These findings led us to hypothesize that T1w/T2w ratio images might be useful to examine intracortical myelin‐related pathological changes in SZ subjects. Furthermore, this method could also be used to estimate myelin‐related changes in WM as a supplementary tool to existing parameters, such as fractional anisotropy (FA) in DTI.

Although it is not yet known to what extent the T1w/T2w ratio value relates to myelin content, the results of previous studies indicated that the signal intensity interindividual difference (mainly due to receiver‐coil bias) was significantly reduced in the ratio image. Furthermore, the signal intensity value could be useful to assess various pathological changes following comparison with the normal tissue signal intensity. This method (i.e., measuring T1w/T2w image signal intensity) has several potential advantages over size measurements (i.e., GM volume and thickness) and DTI. First, signal intensity changes in the SZ brain may be a more sensitive measurement than size changes, as evidenced by findings in various brain diseases, such as infarction and multiple sclerosis. Second, the ratio image intensity measurement can be used for both GM and WM regions and does not depend on anatomical nerve fiber orientation. Thus, this technique could be used to estimate whole‐brain intracortical myelin‐related changes, which are difficult to measure directly using existing noninvasive neuroimaging tools. Furthermore, the ratio image is created by T1w and T2w images, and no additional imaging acquisition time is necessary.

Obviously, the quality and merit of T1w/T2w ratio image increases as the precision of T1w and T2w image co‐localization increases. Indeed, the first report (Glasser and Van Essen 2011) used a sophisticated co‐localization method (i.e., surface‐based registration) and revealed subtle differences in GM signal intensity. We show here that the T1w/T2w ratio image is useful, at least in clinical application, even with a conventional co‐localization method (i.e., voxel‐based registration) that has been used for many functional MRI studies. Thus, we hope that our findings will increase the opportunity for T1w/T2w ratio image application in assessing pathological brain changes in clinical studies, as well as in daily practice.

In this study, we first investigated the global tendency of image signal intensity changes in SZ subjects. If the myelin content reduction is not restricted to specific regions but is widespread in the SZ brain, we would see significantly lower mean T1w/T2w ratio intensity values in the GM and WM of SZ subjects. We found that this was the case; further, the mean WM value was related to the Global Assessment of Functioning (GAF) score, suggesting that the lower signal intensity value corresponds to functional impairment in SZ subjects. These results motivated us to investigate which brain regions were most impaired in the SZ brain. Thus, we used voxel‐wise analysis to identify brain regions in which the T1w/T2w ratio intensities significantly differed between SZ and HC subjects. We found that the T1w/T2w ratio signal intensity in the GM was significantly lower in the right ventral putamen of SZ subjects compared with that of HCs.

## Methods

This study was carried out in accordance with the Declaration of Helsinki and was approved by the Ethics Committee of Wakayama Medical University. Written informed consent was obtained from all subjects.

### Subjects

We examined 29 subjects with SZ (16 males and 13 females) and 33 HC subjects (20 males and 13 females). SZ subjects, diagnosed based on the Diagnostic and Statistical Manual of Mental Disorders, Fourth Edition (DSM‐IV), were recruited from outpatients at Wakayama Medical University Hospital and Wakayama Prefectural Mental Health Care Center. Age‐matched HC volunteers were also recruited from these two institutions.

All subjects were given a standard medical examination before they were recruited. All subjects were deemed healthy and did not have any systemic or neurological illness, mental retardation, or head injury experience. Table 1 shows demographic data, including neuropsychological scales and clinical characteristics.

**Table 1:** Demographic and clinical characteristics

	SZ	HC	Group difference *P*‐value (test)
Age, years	41.4 ± 9.8	37.6 ± 9.8	0.135 (*t‐*test)
Gender (M/F)	16/13	20/13	0.665 (*χ* ^2^ test)
Handedness (R/L)	27/2	33/0	0.126 (*χ* ^2^ test)
Duration of illness, years	17.1 ± 11.3	–	–
JART‐J scores	96.7 ± 11.0	108 ± 8.3	0.0003 (ANOVA)
PANSS_T	64.4 ± 21.7	–	–
PANSS_P	14.4 ± 5.60	–	–
PANSS_N	17.4 ± 6.00	–	–
PANSS_G	32.7 ± 12.0	–	–
GAF	58.2 ± 14.4	–	–
CPZ dose equivalence, mg	629 ± 472	–	–
SGAs	23^a^	–	–
FGAs	8^b^	–	–
Mood stabilizers	5^c^	–	–
Antidepressants	6^d^	–	–
Benzodiazepines	19^e^	–	–

^a,b,c,d,e^Number of subjects taking SGAs, FGAs, mood stabilizers, antidepressants, and benzodiazepines, respectively.

JART‐J, Japanese Adult Reading Test; PANSS, Positive and Negative Syndrome Scale; T, total score; P, positive; N, negative; G, general; GAF, Global Assessment of Functioning Scale; CPZ, chlorpromazine; SGAs, second generation antipsychotics; FGAs, first generation antipsychotics.

### Clinical ratings

Experienced psychiatrists clinically rated SZ subjects prior to MRI acquisition. Symptom severity was assessed using the Positive and Negative Syndrome Scale (PANSS) to rate schizophrenic symptom severity (Kay et al. 1987). Premorbid intelligence quotient (IQ) was estimated using The Japanese Adult Reading Test (JART‐J) with 25 Japanese ideographic script (Kanji) compound words (Matsuoka et al. 2006), which is the Japanese version of the National Adult Reading Test (Nelson 1982). We employed the GAF to assess the daily living status level of SZ subjects. Daily doses of antipsychotics, including depot antipsychotics, were converted to chlorpromazine equivalents using published guidelines (Inagaki and Inada 2006, 2008). Handedness was measured with the Edinburgh Inventory (Oldfield 1971). One SZ subject could not complete the JART‐J.

### MRI acquisition

Scans were performed using a 3.0‐Tesla MRI (Philips, Amsterdam, the Netherlands) with a 32‐channel head coil (SENSE‐Head‐32CH). The mechanical sounds produced during the MRI procedure were minimized by the use of both earplugs and headphones. T1w images were acquired using a 3D T1w Fast Field Echo (FFE): TR, 7.0 ms; TE, 3.3 ms; FOV, 220 mm; matrix scan, 256; slice thickness, 0.9 mm; and flip angle, 10°. T2w images were acquired using a 2D Turbo Spin Echo (TSE): TR, 4681 ms; TE, 80 ms; FOV, 220 mm; matrix scan, 512; slice thickness, 4.5 mm; and flip angle, 90°. The voxel size of the acquired T1w and T2w images was 0.764 × 0.764 × 0.90 mm and 0.275 × 0.275 × 4.5 mm, respectively. Precautions were taken to minimize subjects’ motion during the MRI study by instructing subjects to remain as still as possible. Every scan was checked for image artifacts and gross anatomical abnormalities by a neurologist.

### MRI processing

Magnetic resonance image processing was performed using the SPM8 software package (http://www.fil.ion.ucl.ac.uk/spm; Wellcome Department of Imaging Neuroscience, London, UK) and in‐house software developed with MATLAB (MathWorks, Natick, MA).

#### T1w/T2w ratio image processing

Spatial normalization of T1w images was achieved using a 12‐parameter affine transformation to the International Consortium for Brain Mapping (ICBM) T1 imaging template in SPM8, and each image was resampled to 2‐mm isotropic voxels using the third degree B‐spline interpolation method. Normalized T1w images were segmented into GM and WM images using SPM8, with a probability threshold of 90%. T2w images were also normalized with the T2 image template and were registered to the normalized T1w images using SPM8. We used the third degree B‐spline interpolation method to resample the image to 2‐mm voxels and to reduce the partial volume effect among WM, GM, and cerebrospinal fluid (CSF) (Glasser and Van Essen 2011; Glasser et al. 2014). The T1w/T2w ratio image was created by dividing the signal intensity of the normalized T1w image by the signal intensity of the co‐registered T2w image at each anatomical location. The GM and WM components of the T1w/T2w ratio image were extracted using the segmented normalized T1w image. Both GM and WM T1w/T2w ratio images were then smoothed with an 8‐mm full‐width at half maximum (FWHM) Gaussian kernel. We created both GM and WM binary mask images using the SPM Masking Toolbox (Ridgway et al. 2009). Those mask images were used to perform statistical analyses with SPM8. Furthermore, we created mean GM T1w/T2w ratio image maps for both SZ and HC subjects, and investigated whether they corresponded to the known myelin map pattern (Glasser and Van Essen 2011; Grydeland et al. 2013; Shafee et al. 2015).

To assess the usefulness of the T1w/T2w ratio image, we compared the results with those from a T1w image. We used the intensity inhomogeneity correction tool implemented in SPM8 to correct for scanner bias fields. We set the smoothing and the regularization parameters equal to 60 mm FWHM and 10^−4^, respectively. Then we normalized those bias‐corrected T1w images to the ICBM T1 imaging template in SPM8, and each image was resampled to 2‐mm isotropic voxels using the trilinear interpolation method. Furthermore, we created both GM and WM binary mask images using the SPM Masking Toolbox (Ridgway et al. 2009). Those mask images were used to perform statistical analyses with SPM8.

#### Voxel‐based morphometry image processing

We used voxel‐based morphometry (VBM) to determine if the signal intensity difference of the T1w/T2w image was simply due to brain volume reduction (atrophy) in the SZ group. We used the SPM8 tool, DARTEL (Ashburner 2007), to apply the VBM analysis to the T1w image. We segmented the T1w image into GM, WM, and CSF images using a unified tissue‐segmentation procedure after application of the image‐intensity nonuniformity correction. These segmented GM and WM images were spatially normalized to the customized template in the standardized anatomical space using DARTEL. To preserve the GM and WM volumes within each voxel, we modulated the images using Jacobean determinants derived from the DARTEL spatial normalization, and then smoothed them using an 8‐mm FWHM Gaussian kernel. The final voxel size was 1.5 × 1.5 × 1.5 mm. Note that this analysis procedure was different from that used for the T1w/T2w ratio images, in that the volume information in each voxel was preserved. We created GM binary mask images using the SPM Masking Toolbox (Ridgway et al. 2009). Those mask images were used to perform statistical analyses with SPM8.

### Statistical analysis

#### Demographic and clinical data

Demographic and clinical differences between groups, including age, gender, handedness, and premorbid IQ (assessed by the JART‐J), were examined using the chi‐square test or two‐sample *t*‐tests.

#### Mean GM and WM T1w and T1w/T2w ratio image signal intensity

For each subject, GM and WM mask images were used to separately calculate mean T1w and T1w/T2w ratio image signal intensity values across all GM or WM segment voxels. Then an analysis of covariance (ANCOVA) was performed to assess difference between HC and SZ group values, with age, gender, handedness, and JART‐J as nuisance covariates. Furthermore, we performed a partial correlation analysis with Spearman's method to assess the relationship between the mean values (for T1w and T1w/T2w in GM and WM) and clinical parameters, such as duration of illness, PANSS, GAF, dose of medication with age, gender, handedness, and JART‐J as nuisance covariates. Multiple comparison correction was performed by setting the false discovery rate (FDR) threshold at *P* < 0.05.

#### Regional T1w and T1w/T2w ratio image‐intensity differences

We aimed to identify brain regions with T1w or T1w/T2w ratio image intensity values that were significantly different between HC and SZ subjects; thus, two‐sample *t*‐tests were performed on the respective brain regions in a voxel‐wise manner using GM and WM mask images. Age, gender, handedness, WM volume, and CSF volume were used as nuisance covariates. Although WM and CSF volumes did not significantly differ between the HC and SZ subjects (see Table 4), we included these volumes in the analysis because there was a trend in WM and CSF volumes being smaller and larger in SZ than HC, respectively. We then employed a multiple comparison correction using Gaussian random field theory (see Brett et al.): clusters were considered to be statistically significant when they fell below a cluster‐corrected family‐wise error (FWE) threshold set at *P* < 0.05.

For brain regions that significantly differed between the SZ and HC subjects, we next studied the relationship between the mean signal intensity in those regions along with various clinical parameters, such as duration of illness, dose of medication, and symptom scores, using the multiple regression analysis in SPM8.

#### Brain volume

Group differences in global brain volumes (total GM volume, total WM volume, CSF, and intracranial volume) were assessed using two‐sample *t*‐tests conducted using SPM8. We used DARTEL to identify GM regions whose volumes significantly differed between SZ and HC subjects. Two‐sample *t*‐tests were employed using the mask image, with age, gender, and intracranial volume as nuisance covariates. Multiple comparison correction was performed by setting the FWE at a threshold of *P* < 0.05.

## Results

### Demographic and clinical data

Table 1 shows demographic and clinical information. Only the JART‐J scores significantly differed (*P* = 0.0003); all other scores were similar between HC and SZ subjects.

### The mean T1w/T2w ratio image for HC and SZ subjects

**Figure 1:**
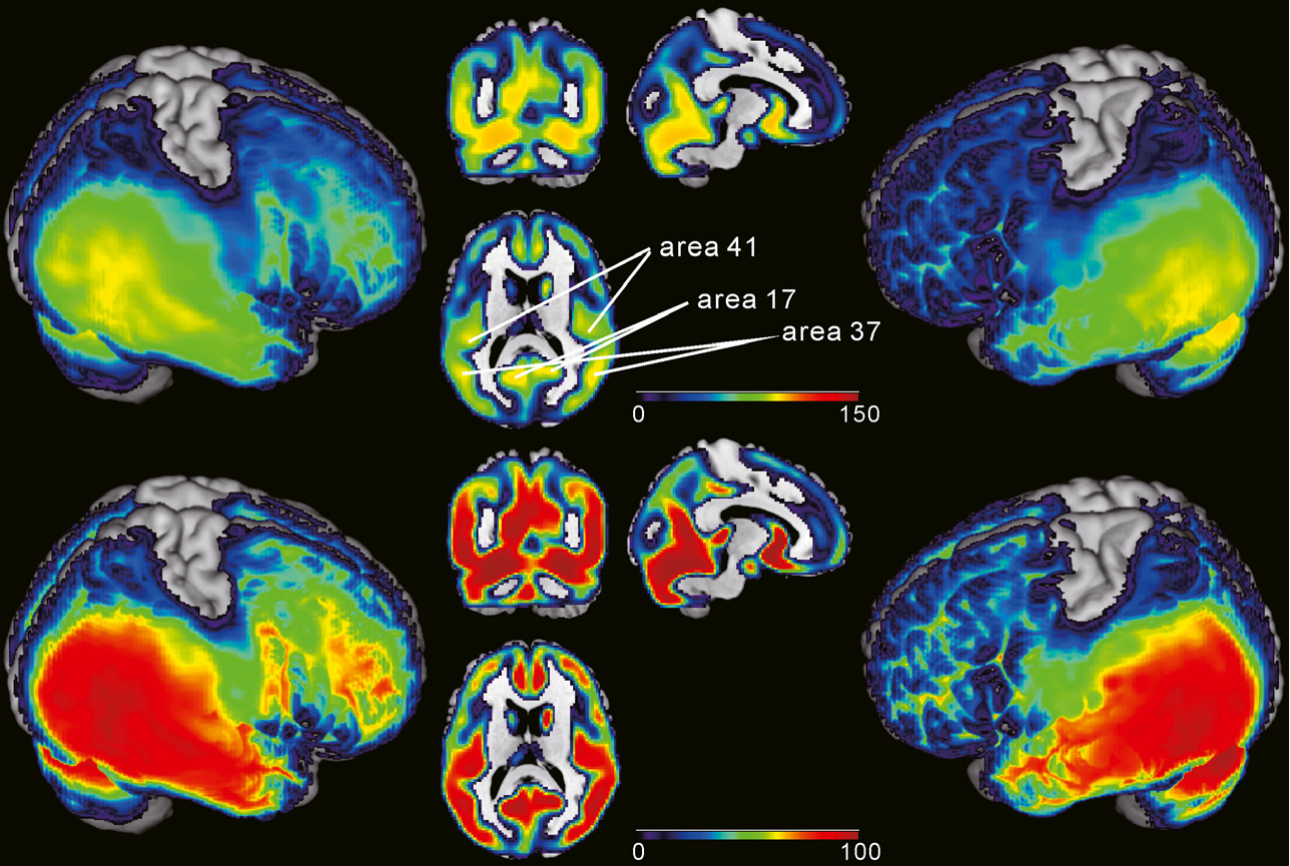
Mean T1‐weighted (T1w)/T2‐weighted (T2w) ratio image for healthy controls (HCs). Bottom: Color maps overlayed on the standard brain represent the signal strength distribution with 0–100th percentile scale. Relatively high signal intensity was distributed around the temporal lobe, cerebellar cortex in both hemispheres. A large part of the sensorimotor areas is missing due to the segmentation procedure (see text in Results). Top: the same color maps are shown with different scale (0–150 percentile) to show relative difference in the occipito‐temporal regions. It is now clearly seen that the primary auditory area (Brodmann area 41), the primary visual area (Brodmann area 17), and V5/MT (Brodmann area 37/19) have high signal intensity compared to the adjacent areas.

Figure 1 (bottom) shows the mean T1w/T2w ratio images for HC subjects with a color map assigned on the basis of the 0 and 100th percentile values. Sensorimotor areas were removed from this map because voxels in the large part of them were lost with our GM segmentation method. The map shows relatively high signal intensity distribution in the temporal lobes, cerebellar cortex and the visual cortex (medial occipital cortex). Because sensorimotor areas are heavily myelinated according to the previous study (Glasser and Van Essen 2011) and were removed from our T1w/T2w ratio map, the next highly myelinated regions such as temporal regions had the highest percentile values in our map compared to the previous report (Glasser and Van Essen 2011). So we presented the same maps with different scale (0–150th percentile) in Figure 1 (top) to show the detailed signal intensity difference within the occipito‐temporal areas. This scale was chosen just to show the relative signal intensity difference around the regions of interest. As seen, the primary auditory areas (area 41), the primary visual areas (area 17), and V5/MT (area 37/19) are now seen to be higher intensity areas compared to the adjacent areas. The mean map for SZ subjects were similar to that for HC (not shown).

Large part of sensorimotor areas were removed from all T1w/T2w ratio signal intensity analyses, which were determined by the GM binary mask images created by the SPM Masking Toolbox (Ridgway et al. 2009).

### Mean GM and WM T1w and T1w/T2w ratio image signal intensity

**Figure 2:**
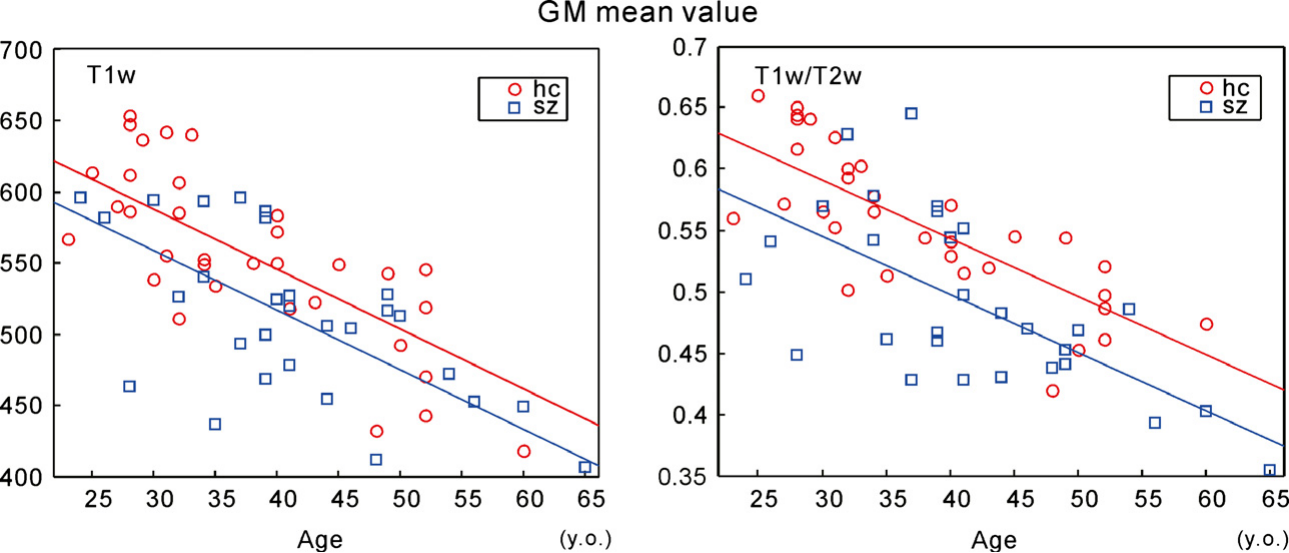
Mean gray matter (GM) signal intensity for each subject. Both the T1w and T1w/T2w ratio image signal intensity values in GM were inversely related with age. The values were lower in the schizophrenia (SZ) group compared with the healthy control (HC) group, particularly for the T1w/T2w ratio image. Each group's linear regression line is shown with the same color. For the detailed statistical analysis results (Table 2).

Figure 2 shows the mean bias‐corrected T1w and T1w/T2w ratio image signal intensity values among all GM voxels for all the subjects. As shown, both values were inversely related with age for both the SZ and HC groups. An ANCOVA (with age, gender, handedness, and JART‐J as nuisance covariates) showed that the T1w/T2w ratio image signal intensity values significantly differed between the HC and SZ groups [*F* (1, 55) = 10.883, *P* = 0.002]; however, no significant differences were detected in the bias‐corrected T1w signal analysis (Table 2).

**Table 2:** The result of ANCOVA with age, gender, handedness, and JART as nuisance covariates

	Gray matter				White matter			
	T1w/T2w		T1w		T1w/T2w		T1w	
	*F*‐ratio	*P*	*F*‐ratio	*P*	*F*‐ratio	*P*	*F*‐ratio	*P*
ANCOVA	10.883	0.002	3.621	0.062	8.183	0.006	2.107	0.152
Covariates								
Age	57.627	<0.0001	73.581	<0.0001	4.397	0.041	0.124	0.726
Gender	6.421	0.014	13.077	0.001	6.021	0.017	10.172	0.002
Handedness	1.003	0.321	8.977	0.004	1.361	0.248	0.324	0.572
JART‐J	0.116	0.735	0.398	0.531	0.718	0.4	1.705	0.197

JART‐J, Japanese Adult Reading Test.

**Figure 3:**
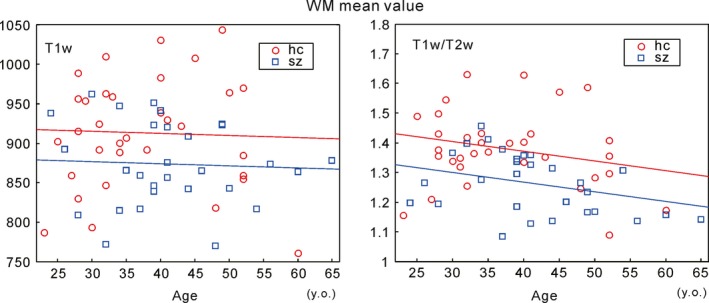
Mean white matter (WM) signal intensity for each subject. The T1w/T2w image signal intensity values in WM were inversely related with age in both the schizophrenia (SZ) and healthy control (HC) groups; values were lower in the SZ group than those observed in the HC group, similar the trend observed in the gray matter (GM) values (see Fig. 2). Each group's linear regression line is shown with the same color. We observed a similar tendency in the T1w image values; however, the tendency was less pronounced than that observed in the T1w/T2w image. For the detailed statistical analysis results (Table 2).

Figure 3 shows the mean bias‐corrected T1w and T1w/T2w ratio image signal intensity values among all WM voxels for all subjects. The ANCOVA (age, gender, handedness, and JART‐J as nuisance covariates) revealed a significant difference in the T1w/T2w ratio signal intensity between the HC and SZ groups [*F* (1, 55) = 8.183, *P* = 0.006]. No significant differences were detected in the bias‐corrected T1w signals (Table 2).

Table 3 shows the partial correlation analysis results between the mean image signal intensity values and clinical parameters, excluding the effect of age, gender, handedness, and JART‐J. Multiple comparisons were performed by setting the FDR threshold at *P* < 0.05. The mean WM T1w/T2w ratio intensity was significantly correlated with the PANSS_P and GAF scores. A similar trend was observed between the mean GM T1w/T2w ratio intensity and clinical parameters (i.e., PANSS_N and GAF); however, the trend was not statistically significant.

**Table 3:** Partial correlations between signal intensity and clinical parameters

	T1w/T2w			T1w		
	*ρ*	*P*_corr	*P*_uncor	*ρ*	*P*_corr	*P*_uncor
Gray matter						
Duration of illness, years	−0.144	0.502	0.502	−0.0302	0.889	0.889
PANSS_P	−0.344	0.150	0.100	−0.0676	0.889	0.754
PANSS_N	−0.473	0.0586	**0.0195**	−0.171	0.889	0.423
PANSS_G	−0.407	0.0967	**0.0483**	−0.127	0.889	0.555
GAF	0.511	0.0586	**0.0106**	0.147	0.889	0.494
CPZ dose equivalence	−0.293	0.198	0.165	0.0432	0.889	0.841
White matter						
Duration of illness, years	−0.147	0.493	0.493	−0.0713	0.971	0.740
PANSS_P	−**0.523**	**0.0389**	**0.00871**	0.00775	0.971	0.971
PANSS_N	−0.385	0.127	0.0634	0.0468	0.971	0.828
PANSS_G	−0.338	0.128	0.106	0.0405	0.971	0.851
GAF	**0.500**	**0.0389**	**0.0130**	−0.120	0.971	0.575
CPZ dose equivalence	−0.349	0.128	0.0943	0.127	0.971	0.555

*P*_corr, FDR corrected *P*;* P*_uncor, uncorrected *P*. Bold characters indicate p values < 0.05.

PANSS, Positive and Negative Syndrome Scale; T, total score; P, positive; N, negative; G, general; GAF, Global Assessment of Functioning Scale; CPZ, chlorpromazine; T1w, T1‐weighted; T2w, T2‐weighted.

### Voxel‐wise analysis of T1w and T1w/T2w ratio image signal intensity

**Figure 4:**
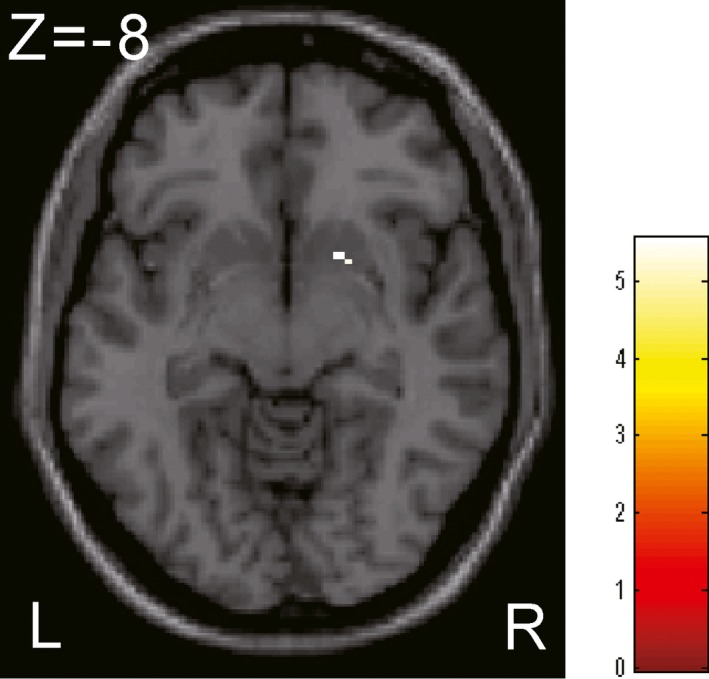
Gray matter (GM) regions in the schizophrenia (SZ) brain where the T1w/T2w ratio image signal intensity values were significantly different from those in healthy control (HC) subjects. The right ventral putamen (including the nucleus accumbens) was identified using a voxel‐wise two‐sample *t*‐test (*P* family‐wise error [family‐wise error, FWE] < 0.01). See Table 4 for the Montreal Neurological Institute (MNI) coordinates of the region. Scale: *t* score.

**Figure 5:**
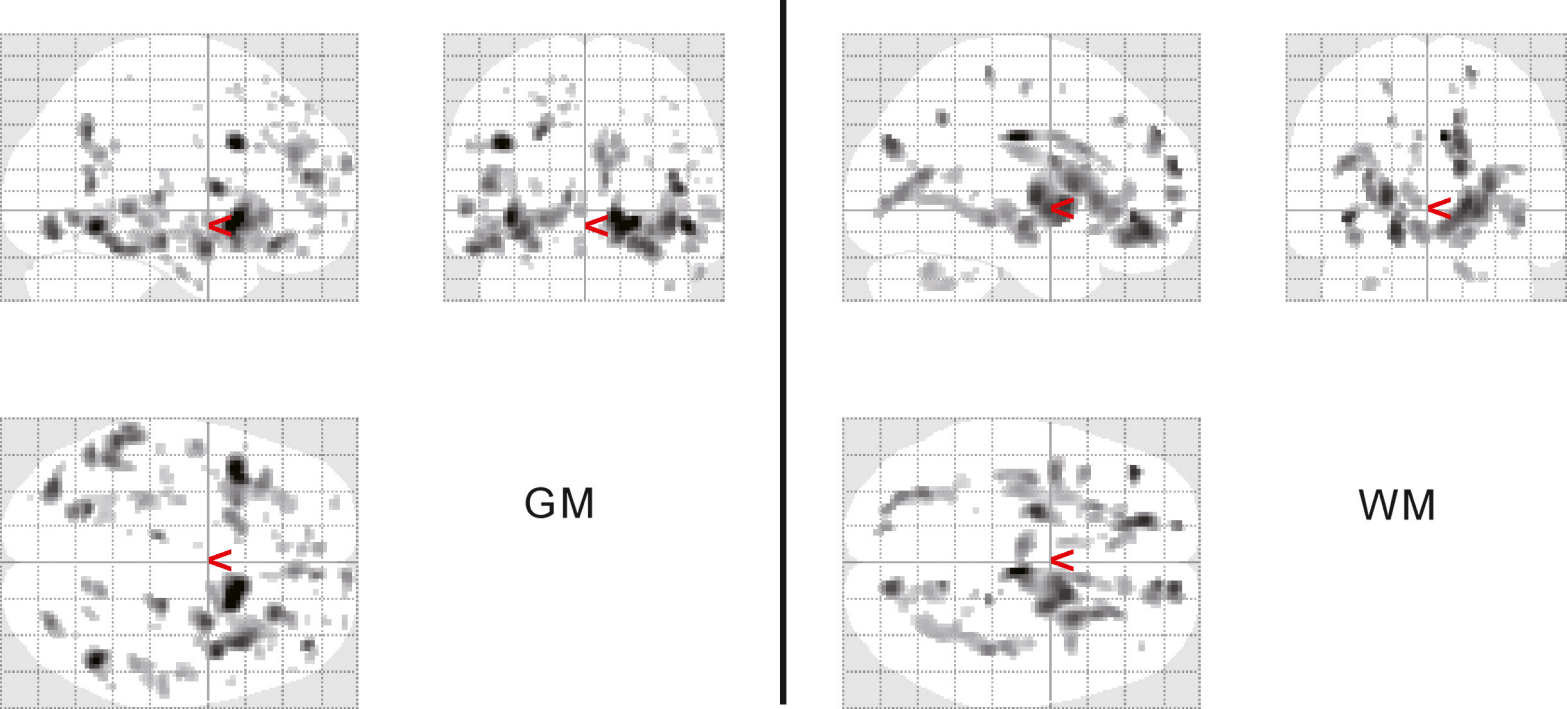
Distribution of the regions with relatively high signal intensity difference in T1w/T2w image between healthy control (HC) and schizophrenia (SZ). Regions with significantly different signal intensity (uncorrected *P *<* *0.001 and cluster size threshold = 0) between HC and SZ are shown for Gray matter (GM) (left) and white matter (WM) (right). Age, gender, handedness, WM volume, and cerebrospinal fluid (CSF) volume were included as nuisance covariates. As seen, in both GM and WM, the regions were distributed widely in the brain but rather accumulated in the ventral frontal lobes and the temporal lobes.

The bias‐corrected T1w signal intensity was similar between the HC and SZ groups for both GM and WM. Figure 4 shows the region in which the GM T1w/T2w ratio signal intensity values in the SZ group were significantly lower than those in the HC group (*P* FWE < 0.05), the right ventral putamen (including the nucleus accumbens) (see Table 4). The mean signal intensity value in these regions was not related to any of the clinical parameters, such as illness duration, dose of medication, or various symptom parameters. Although only a restricted region showed the significant difference between HC and SZ, there were many regions subsignificantly different between HC and SZ throughout the whole brain, which is shown in Figure 5 (uncorrected *P* < 0.001).

**Table 4:** GM regions where significant differences were found in the T1w/T2w ratio image between HC and SZ subjects

Identified Regions	MNI (mm)			Peak FWE	Cluster FWE	*T*	*Z*	Cluster size
	*x*	*y*	*z*	Corrected *P*	Corrected *P*			
Right ventral putamen	20	10	−8	0.024	0.014	5.55	4.92	4

GM, gray matter; SZ, schizophrenia; HC, healthy control; MNI, Montreal Neurological Institute; FWE, family‐wise error.

### VBM analyses

The mean total GM and WM volumes in the SZ brain were 693.4 ± 84.4 and 516.9 ± 65.0 mm^3^, respectively, which were not significantly different from the mean HC group values (Table 5). The CSF volume and the intracranial volume were not significantly different from those of the HC group. Furthermore, we did not find any region in the SZ brain (GM) in which the volume was significantly different from the HC group (*P *> 0.05, FWE‐corrected).

**Table 5:** Brain volume differences between HC and SZ subjects

	SZ	HC	Group difference *P*‐value (test)
Total gray matter volume (mm^3^)	693.39 ± 84.43	712.68 ± 60.93	0.302 (*t‐*test)
Total white matter volume (mm^3^)	516.86 ± 65.03	538.90 ± 49.47	0.136 (*t‐*test)
Cerebrospinal fluid (mm^3^)	359.34 ± 41.50	344.44 ± 47.70	0.198 (*t‐*test)
Intracranial volume (mm^3^)	1569.60 ± 180.16	1596.00 ± 149.12	0.632 (*t‐*test)

SZ, schizophrenia; HC, healthy control.

## Discussion

In this study, we found that the T1w/T2w ratio image could be used to detect SZ brain abnormalities with greater sensitivity than that of T1w images. T1w signal intensity is mainly related to myelin content in both GM and WM (Lutti et al. 2014; Stuber et al. 2014b). Dividing the T1w signal intensity by the T2w signal intensity further increases the contrast of the myelin content and reduces the signal receiver‐coil bias (Glasser and Van Essen 2011). As a result, the T1w/T2w ratio image successfully reveals signal intensity changes with age in both the brain and spinal cord (Grydeland et al. 2013; Teraguchi et al. 2013).

### Mean T1w/T2w ratio image

As shown in Figure 1, relatively high T1w/T2w ratio signal intensity areas were observed in the temporal lobe, and cerebellar cortex in both hemispheres. The large part of the sensorimotor areas is missing because of the GM template we made. To compare the map with previous results (Glasser and Van Essen 2011; Grydeland et al. 2013; Shafee et al. 2015), we changed the scale to 0–150 percentile to check the detailed signal intensity difference in the occipito‐temporal areas. Because the signal intensity is the highest in the sensorimotor areas which is missing in our map, signal intensity in most of the sensorimotor areas must be more than 90 percentile for the previous studies. But for our map, it is in the temporal lobe. This is why the large part of the temporal lobe has higher signal intensity in our map. In the same map with 0–150 percentile scale, we could see the relatively high signal intensity areas in the primary auditory area (area 41), the primary visual area (area 17), and MT/V5 (area37/19). Thus, T1w/T2w ratio intensity distribution pattern is roughly consistent with previous reports (Glasser and Van Essen 2011; Grydeland et al. 2013; Shafee et al. 2015), indicating that our T1w/T2w ratio image represented myelin content despite the voxel size differences between T1w and T2w images.

### Pathological meaning of the T1w/T2w ratio signal intensity reduction in the SZ brain

We believe that the reduced T1w/T2w ratio signal intensity values found in the SZ brain in this study are related to myelination deficits for the following reasons. First, the T1w/T2w ratio image signal intensity in the normal human brain correlates quite well with subcortical myelin development (Glasser and Van Essen 2011; Grydeland et al. 2013); T1w signal intensity is mainly related to myelin content in both GM and WM (Lutti et al. 2014; Stuber et al. 2014a). Second, a reduced DTI FA value in the SZ brain was found in various WM fiber bundles (e.g., arcuate fasciculus, uncinated fasciculus, etc.) (Kubicki et al. 2007; Ellison‐Wright and Bullmore 2009) that connect various GM regions, including the ventral putamen (identified in this study). The WM myelination impairment revealed by DTI should have a close relationship with GM myelination because GM myelin content is generally related to the subcortical WM myelin content (Glasser and Van Essen 2011); some exceptional cases may exist, however (Tomassy et al. 2014). Furthermore, T2 relaxation time was prolonged in both GM and WM in SZ subjects (Pfefferbaum et al. 1999), corresponding to myelin loss. Third, no other macroscopic pathological changes that could affect MRI signal intensity has been observed in the SZ brain, such as abnormal iron deposition or gliosis (Iritani 2007; Takahashi and Sakurai 2013). It is assumed that GM dendritic loss (Bennett 2011) and increased WM interstitial neuron number (Akbarian et al. 1996; Kirkpatrick et al. 1999, 2003) do not affect MRI signal intensity. Fourth, recent genetic studies revealed myelin‐ and oligodendrocyte‐related genetic abnormalities in the SZ brain (Hakak et al. 2001; Dean et al. 2009; Roussos et al. 2012; Roussos and Haroutunian 2014). Fifth, GM atrophy (volume loss) would simply not account for the low T1w/T2w ratio signal intensity observed in the SZ brain. Our DARTEL VBM analysis did not reveal any regions that were significantly different between the HC and SZ subjects. Although this might be due to the relatively small number of subjects examined in this study, our results indicate that the volume reduction was not simply related to the T1w/T2w image signal intensity. Moreover, the nucleus accumbens volume in the SZ subjects, where we found a significant signal intensity decrease (see Fig. 4 and Table 4), was reportedly increased (i.e., the right side was more prominent than the left side) in SZ subjects compared with the HC subjects (Lauer et al. 2001).

### Global T1w/T2w ratio signal intensity differences

The mean signal intensity values across all GM and WM voxels were significantly reduced in the SZ brain (Figs. 2 and 3). This indicates that SZ brain abnormalities are widely distributed in the whole brain in contrast to the previous idea that abnormalities exist in restricted regions, such as the prefrontal area. Although our voxel‐wise analysis revealed only the small part of the right ventral putamen, subsignificant highly different signal intensity between the groups are seen in the wide brain areas (Fig. 5). Furthermore, the WM mean T1w/T2w ratio intensity value was significantly related to the GAF score in SZ subjects; the GM mean T1w/T2w ratio intensity showed a similar trend. The GAF rates social functioning (Bengtsson‐Tops and Hansson 1999), which is related to the ability to integrate many kinds of cognitive functions in accordance with various social situations. The widespread reduction in myelin content in the brain may be related to global brain function [e.g., as shown by global neural synchronization (Billeke et al. 2015)]. This might explain why we did not detect a significant relationship between the mean signal intensity value in the right ventral putamen (i.e., the signal intensity of which was significantly reduced in the SZ group) and the clinical parameters.

In line with this consideration, intraindividual variability (IIV) of reaction time is related to GM myelin content in broad cortical regions (Grydeland et al. 2013) and WM microstructures (Fjell et al. 2011; Tamnes et al. 2012) of healthy subjects. The IIV is a measure of individual performance consistency, which reflects various aspects of cognitive function. Although the previous study indicated that the left prefrontal and inferior parietal region volumes were related to the GAF score (Wilke et al. 2001), the volume was not significantly different from that of the HC group, suggesting that the volume reduction in these regions alone does not cause a decrease in the GAF score.

The mean SZ WM T1w/T2w ratio signal intensity was negatively correlated with the PANSS_P score (Fig. 3). This is consistent with previous studies showing that the global FA in WM is negatively related to the PANSS_P score (Skelly et al. 2008; Knochel et al. 2012), in that both measurements reflect WM myelination. Our data also support a “disconnection hypothesis” of positive symptoms in SZ (Friston 1998), which indicate that aberrant WM connectivity is the structural basis of psychotic symptoms.

### Regional T1w/T2w ratio signal intensity differences

In addition to the global signal intensity analysis, we performed a voxel‐wise analysis to examine regional abnormalities in the SZ brain. We found that GM T1w/T2w ratio signal intensity in the SZ group was significantly reduced in the right ventral putamen (including the nucleus accumbens) (Fig. 4), whereas no significantly different regions were detected in the bias‐corrected T1w image. These results again indicate that the T1w/T2w ratio image is more sensitive to pathological changes in the SZ brain than is the T1w image. Although global T1w/T2w ratio signal intensity difference in GM between HC and SZ was observed as shown in Figures 2 and 3, regional voxel‐wise analysis identified significantly different region only in the right ventral putamen. We found, however, subsignificant low signal intensity areas are widely distributed in the brain (Fig. 5). One reason why those areas did not reach the multiple comparison corrected significance level could be due to the relatively small number of subjects investigated. The other reason might be due to the lower spatial resolution of the T2w image compared to that of T1w image, which resulted in the poor identification of individual GM voxels due to the partial volume effect.

Putamen volume is reportedly increased in most studies on chronic SZ subjects, (Iosifescu et al. 1997; Mamah et al. 2007; Brandt and Bonelli 2008), as is the nucleus accumbens (Lauer et al. 2001). It was hypothesized that positive symptoms of SZ, such as auditory hallucination (AH), are induced by hyperactivity of the dopaminergic pathway, which projects from the ventral tegmental area to the ventral striatum (Meltzer and Stahl 1976). This is known as the mesolimbic hypothesis. The nucleus accumbens is the main target of this projection (Crow et al. 1977). In vivo neuroimaging studies reported: (1) stronger connectivity between the putamen and inferior frontal gyrus of SZ subjects with AH than those without AH (Hoffman et al. 2011); (2) a significant correlation between the vividness of AH and the activity of the nucleus accumbens in SZ (Raij et al. 2009); and (3) a relationship between the functional connectivity of the nucleus accumbens and other mesolimbic structures and increased numbers of hallucination modalities (Rolland et al. 2014). Taken together, the ventral putamen and the nucleus accumbens are thought to play a pathophysiologically important role in SZ. Our results suggest the existence of a myelin deficit in these regions in addition to pathological changes that increase the volume, and that both changes may cause such SZ‐related dysfunction.

### VBM analyses

A number of studies have consistently shown that several brain regions in patients with SZ are significantly smaller than those in HCs (Kuroki et al. 2006; Ellison‐Wright et al. 2008). We were unable to reproduce these results in our study, likely because of the rather small number of subjects used; however, we observed similar tendencies in the GM, WM, CSF, and intracranial volumes that corresponded with previous reports (see Table 5). These results suggest that T1w/T2w image intensity more sensitively detects abnormalities in the SZ brain than VBM, likely due to its larger effect size. T1w/T2w image signal intensity reduction may occur before the brain volume reduction is detectable.

### Limitations

There are some limitations to our study. First, the spatial resolution of T2w image used in this study was different from that of the T1w image (especially slice thickness of 4 mm for T2w image is much larger than that for T1w), which may have reduced the merit of the T1w/T2w image (e.g., myelin‐related contrast increase and reduction of receiver‐coil bias). Furthermore, the partial volume effect must be higher in T2w image, which might be the reason why we did not see any difference in the signal intensity of GM as discussed above. However, as mentioned in Results section, the T1w/T2w ratio signal intensity map was roughly consistent with the myelin map pattern (Glasser and Van Essen 2011; Shafee et al. 2015), in spite of lack of voxels in sensorimotor areas in our study. This finding supports that the co‐registration between the T1w and T2w images would be performed fairly accurately and that the reduction in the scanner bias fields would also be appropriate. Furthermore, when we performed voxel‐wise two‐sample *t*‐test on the T1w/T2w ratio images, we included WM and CSF volumes as nuisance covariates to reduce the partial volume effect. Higher spatial resolution, however, should improve both the sensitivity and specificity of this measurement.

Second, it remains unclear if our MRI T1w/T2w ratio image acquisition measurements were optimized to elucidate entire brain abnormalities in patients with SZ. Acquisition parameters for T1w and T2w images and magnetic field strength affect the ratio image signal intensity and contrast, as discussed by a previous study (Glasser and Van Essen 2011). Third, some improved T1w/T2w ratio intensity analysis methods have been presented (Ganzetti et al. 2014; Shafee et al. 2015); we, however, did not use such corrections.

Forth, our voxel‐based method to register the T1w image to the corresponding T2w image was less precise than the surface‐based registration method used by Glasser and Van Essen (Glasser and Van Essen 2011). Our voxel‐wise registering method, however, is simple enough to apply the T1w/T2w ratio image to clinical practice. It should be noted that we found T1w/T2w ratio signal intensity abnormalities in the SZ brain. We also confirmed that the sensitivity was markedly increased compared to the T1w image, even if our T1w/T2w image method was not optimized. Thus, if optimized T1w/T2w images are used, we expect that the T1w/T2w image method will detect even more abnormal regions in the SZ brain than those we found in this study.

Finally, all of our subjects with SZ were taking several medications (Table 1) that might have affected the MRI signal intensity (Dazzan et al. 2005; Lieberman et al. 2005). However, we did not find any relationship between the amount of medication (or illness duration) and theT1w/T2w signal intensity.

## Conclusions

We found that the T1w/T2w ratio image revealed SZ brain abnormalities in both GM and WM with higher sensitivity than that of the T1w image, which was likely related to impaired myelination. We believe that the T1w/T2w ratio image could be a useful method to elucidate the pathological basis of SZ symptoms and the developmental processes of the SZ brain.

## Author contributions

YK conceived and designed the experiments. JI, SU, KS, and MT performed the experiments. TI, TD, and YK analyzed the data. TI, JI, YK, SU, and KS wrote the manuscript.

## Conflict of Interest

The authors declare no conflicts of interest.
